# Inhibition of miR-17~92 Cluster Ameliorates High Glucose-Induced Podocyte Damage

**DOI:** 10.1155/2020/6126490

**Published:** 2020-07-21

**Authors:** Xiaobao Fan, Zhiming Hao, Zhenjiang Li, Xiaoming Wang, Jing Wang

**Affiliations:** ^1^Department of Rheumatology and Immunology, The First Affiliated Hospital of Medical College of Xi'an Jiaotong University, Xi'an City, Shaanxi Province 710061, China; ^2^Nephrotic Hemodialysis Center, Shaanxi Provincial People's Hospital, Xi'an City, Shaanxi Province 710068, China

## Abstract

The loss and damage of podocytes is an early feature of diabetic nephropathy (DN). The miR-17∼92 cluster was dysregulated in diabetic and polycystic kidney disease patients, but its role in DN is unclear. Hence, an *in vitro* study on the high glucose- (HG-) treated mouse podocytes (MPC5) was designed to elucidate the effect of miR-17∼92 cluster downregulation on cell viability, apoptosis, inflammation, fibrosis, and podocyte function. The results suggested that the miR-17∼92 cluster members miR-17-5p, miR-18a, miR-19a, miR-19b, miR-20a, and miR-92a were upregulated in the renal biopsy tissue of DN patients and HG-treated MPC5. The downregulation of the miR-17∼92 cluster effectively suppressed the cell apoptosis, inflammation, fibrosis, and podocyte dysfunction in HG-stimulated MPC5 cells. The bioinformatics analysis and rescue experiments showed that ABCA1 (ATP-binding cassette transporter A1) is an effector of the miR-17~92 cluster. Silence of ABCA1 inhibited the protective effect of the miR-17∼92 cluster downregulation on podocyte damage. In summary, this research indicated that the downregulation of the miR-17∼92 cluster ameliorates HG-induced podocyte damage via targeting ABCA1.

## 1. Introduction

Diabetic nephropathy (DN), the most common cause of end-stage renal disease, is a complication of mellitus patients, affected about 20–40% diabetes [1]. It is characterized by the presence of proteinuria, glomerular and tubular glomerular basement membrane thickening, podocyte dysfunction, and inflammation [2]. In clinical practice, the loss of podocytes and impaired podocyte integrity were found in the early stage of diabetes mellitus patients [3]. Podocytes (glomerular visceral epithelial cells) are highly differentiated cells that are mainly responsible for maintaining the glomerular filtration barrier [4]. Podocyte injury leads to the increased glomerular permeability, allowing proteins and other mediators to pass into the tubular lumen, leading to proteinuria and kidney dysfunction [5]. Therefore, the podocyte damage critically contributes to the progression of DN [6].

Emerging evidences have demonstrated that miRNAs participated in the regulation of DN progression through inhibiting posttranscriptional gene expression [7–9]. For example, miR-29c was upregulated in DN and induced cell apoptosis and increased extracellular matrix protein accumulation [10]. However, these studies principally focus on single miRNAs. Following the discovery of miRNA gene clusters, many reports found that miRNAs accomplish their function via working in combination. For instance, miR-143/145 cluster is downregulated in colorectal cancer as well as in some other cancers cell lines, contributing to poor prognosis [11, 12]. In this study, we focused on miR-17∼92, an oncogenic miRNA cluster, composed of seven miRNA members miR-17, miR-18a, miR-19a, miR-19b, miR-20a, and miR-92a [13]. The expression of miR-17-5p, miR-18a, miR-19b, and miR-20a was increased in diabetes patients and positively related to the risk of the type 2 diabetes mellitus and impaired fasting glucose [14–16], while the levels of miR-19a and miR-92a were declined in diabetes and have been reported to correlate with diabetic lower limb ischemia [17, 18]. Besides, miR-17∼92 miRNA cluster is increased and accelerates the kidney cyst growth in a mouse model of polycystic kidney disease [19]. Although, the miR-17∼92 cluster is required for nephron development and normal renal function in mouse embryonic development [20]. However, the function of the miR-17∼92 cluster in the progress of DN remains unclear.

ATP-binding cassette transporter A1 (ABCA1) is a cholesterol exporter, which plays a protective role in cardiovascular disease and diabetes [21]. It has been reported that ABCA1 mutations can decrease plasma high-density lipoprotein levels, augment the risk of type 2 diabetes, and aggravate cardiovascular disease [22]. In addition, ABCA1 was decreased in diabetes mellitus patients and DN patients [23]. Enhanced ABCA1-mediated renal cholesterol efflux could relieve DN; besides, ABCA1 participated in the regulation of inflammation progress in DN patients [24].

Increasing evidence suggested that hyperglycemia contributes to podocyte injury [25]. High glucose could trigger fibrosis, cell apoptosis, and function dysfunction in cultured podocytes [26]. In the present study, *in vitro* study of the high glucose- (HG-) treated mouse podocytes (MPC5) was designed to explore the role of miR-17∼92 cluster downregulation in podocyte damage. Further, the underlying molecular mechanisms of the miR-17∼92 cluster in the regulation of the function of HG-stimulated MPC5 podocytes were explored. Our study indicated that the si-miR-17∼92 cluster plays a protective role in HG-stimulated MPC5 cells through regulating the expression of ABCA1.

## 2. Materials and Methods

### 2.1. Patients and Sample Collection

Thirty-two diabetic nephropathy patients who were admitted to the First Affiliated Hospital of Medical College of Xi'an Jiaotong University between 2015 and 2017 were employed in this study. Their blood samples and 15 renal biopsy samples were collected. Twenty-six healthy controls were included this study; they were confirmed free of chronic diseases, diabetes mellitus, kidney diseases, hypertension, or other serious diseases. In addition, 15 normal renal tissue samples were collected through renal biopsy. The renal specimens were adopted through needle biopsy of kidney, with specimen length 12.3 ± 5.4 mm and mean glomerular number 16.8 ± 6.2 [27]. This study was as approved by the Ethical Committee of the First Affiliated Hospital of Medical College of Xi'an Jiaotong University (2017-150), and informed consent was obtained from all study participants.

### 2.2. Cell Lines

Conditionally immortalized mouse podocytes (MPC5) were provided from Shanghai Institute of Cellular Biology of Chinese Academy of Sciences (Shanghai, China) and cultured as previously described [26]. MPC5 cells were verified without mycoplasma through a MycoProbe Mycoplasma Detection Kit (catalog # CUL001B, R&D Systems). Well-differentiated MPC5 cells were exposed to serum-free RPMI 1640 medium; 25 mM glucose (HG) was added into the culture medium and maintained for 24 h to establish a podocyte damage cellular model. For the control group, MPC5 cells were cultured in RPMI 1640 medium containing 10% fetal calf serum, and 5 mM glucose was added [28].

### 2.3. RNA Extraction and RT-PCR

The miRNAeasy mini kit was used to extract miRNAs following the manufacturer's instructions (Qiagen, Carlsbad, CA). ABCA1 mRNA were isolated by TRIzol reagent (Invitrogen Carlsbad, CA, USA). RT-PCR was performed as previously reported [26]. 1 *μ*g of total RNA was reversely transcribed to cDNA using a TaqMan microRNA Reverse Transcription Kit (Applied Biosystems, F`oster City, CA). RT-PCR was performed using the DyNamo SYBR1 Green qPCR kit (Takara, Japan). The messenger RNA (mRNA) levels of miRNAs were calculated with the 2^−*ΔΔ*Ct^ method, which were normalized to the GADPH cDNA level.

### 2.4. Cell Transfection

Antagomirs of the miR-17∼92 cluster (Anta-miR-17, Anta-miR-18a, Anta-miR-19a, Anta-miR-19b, Anta-miR-20a, and Anta-miR-92a), siRNA of ABCA1 (si-ABCA1), and lentiviral-mediated overexpression vector of ABCA1 (ov-ABCA1) were designed and purchased from Genomeditech Comp. (Shanghai, China). miRNA-17-5p mimics, miR-20a-5p mimics, and control mimics (NC-mimic) were synthesized and purchased from GenePharma (Shanghai, China). Lipofectamine™ 3000 was used to transfect antagomirs of the miR-17∼92 cluster, siRNA of ABCA1, overexpression vector of ABCA1, miRNA-17-5p mimics, miR-20a-5p mimics, and control mimics into cells according to manufacturer's protocol (Invitrogen).

### 2.5. Luciferase Assay

Wild-type and mutant 3′UTR fragments of ABCA1 gene were cloned into pGL3 luciferase reporter vector (Promega, Madison, Wisconsin, WI, USA) to construct dual luciferase reporter vector, named as LUC-WT-ABCA1 and LUC-MUT-ABCA1. MPC5 cells were seeded into 96-well plates and cotransfected with LUC-WT-ABCA1 (LUC-MUT-ABCA1) luciferase reporter and miRNA-17-5p mimics or miR-20a-5p mimics. After transfection for 48 h, Dual-Luciferase Reporter Assay System (Promega) was adopted to examine the Luciferase activity.

### 2.6. Cell Viability

MPC5 were planted into 96-well plates. After treatment, 10 *μ*L CCK-8 regents (Dojindo, Japan) were added into the culture medium and incubated for 3 h. A SpectraMax M5 microplate reader was used to record the absorbance at 450 nm (Molecular Devices, USA).

### 2.7. Western Blotting

Podocytes were collected and lysed by RIPA buffer; then, a high-speed centrifuge (14,000 x g, 4°C, 15 min) was performed, and the supernatant was gathered. The protein samples were quantified by Pierce™ Rapid Gold BCA Protein Assay Kit (Thermo Fisher Scientific). Then, protein was loaded onto SDS-PAGE and transferred to Nitrocellulose Blotting membranes (Millipore, Billerica, MA, USA). After being blocked with 5% skim milk, blotting membranes were incubated with specific primary antibodies overnight at 4°C. At last, HRP-conjugated IgG secondary antibodies were added and incubated at room temperature for 2 h. Chemiluminescent detection reagents (Pierce) was used to visualize the immunoblots, and Image Lab imaging software was adopted to calculate the relative integrated density values. Primary antibodies used in this study are as follows: Rabbit anti-nephrin antibody (Abcam, ab216341), anti-Wilms Tumor Protein (WT-1) antibody (Abcam, ab89901), podocin polyclonal antibody (Invitrogen, A5-80863), Rabbit Anti-Collagen I antibody (Abcam, ab34710), Alpha-Smooth Muscle Actin (a-SMA) Antibody (Invitrogen, MA1-06110), and Fibronectin/FN1 (E5H6X) Rabbit mAb (CST, #26836). Secondary antibodies: Goat Anti-Rabbit IgG H&L (Abcam, ab150077), and Anti-mouse IgG, HRP-linked Antibody (CST, #7076).

### 2.8. Enzyme-Linked Immunosorbent Assay (ELISA)

The levels of IL-6 and TNF-*α* in the culture medium of MPC5 cells were determined using human IL-6 ELISA kit (Abcam, ab178013) and human TNF-*α* ELISA kit (Abcam, ab181421) according to the manufacturer's protocol.

### 2.9. Data Analysis

The data of miR-17~92 luster expression in the plasma and tissue samples of DN patients were presented as mean (SD). Cell experimental data were presented as mean ± SEM. All statistical analyses were performed with Student's *t*-test or ANOVA followed by Dunnett's test through SPSS 20.0 statistical software (SPSS Inc., Chicago, IL, USA). *p* < 0.05 was considered statistically significant. All experiments were repeated at least three times.

## 3. Results

### 3.1. The Level of miR-17∼92 Cluster Members in DN Patients

**Figure 1:**
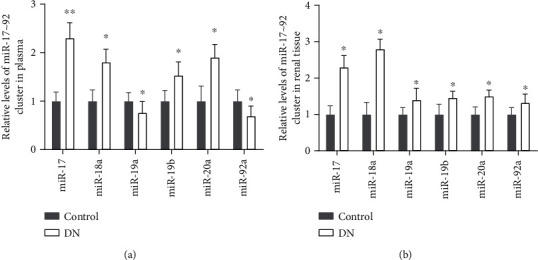
The expression levels of the miR-17 ∼ 92 cluster members in DN patients. (a, b) The levels of the miR-17∼92 cluster members in the plasma (*n* = 32) and renal tissues (*n* = 15) were measured through RT-qPCR, compared with the control group (*n* = 15). DN: diabetic nephropathy, ^∗^*p* < 0.05, vs. the control group.

The relative levels of the miR-17∼92 cluster members in the plasma and kidney tissues were examined through RT-qPCR assay. The results suggested that the expression levels of miR-17-5p, miR-18a, miR-19b, and miR-20a were augmented in the plasma and kidney tissues of DN patients, while the expression of miR-19a and miR-92a was decreased in the plasma but increased in the kidney tissues of DN patients, compared with the control group (Figures 1(a) and 1(b)).

### 3.2. The Effect of miR-17∼92 Cluster Silence on Cell Viability, Apoptosis, and Inflammation of HG-Treated MPC5 Cells

**Figure 2:**
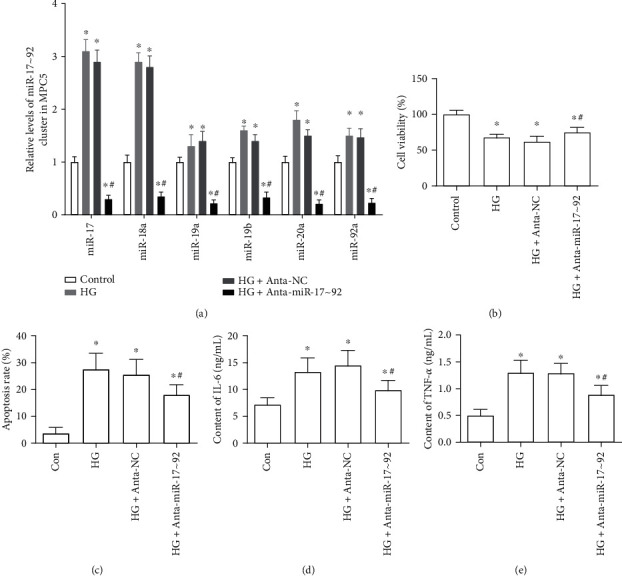
The effect of miR-17∼92 cluster silence on cell viability, apoptosis, and inflammation of HG-treated MPC5 cells. (a) The expression levels of miR-17∼92 cluster members in MPC5 cells. (b, c) Cell viability (b) and apoptosis (c) of HG-treated MPC5 cells were detected through CCK-8 assay and Annexin V/PI double stain assay. (d, e) The content of IL-6 (d) and TNF-*α* (e) in the culture medium of MPC5 cells were measured through ELISA kits. Cells were transfected with antagomirs of the miR-17∼92 cluster members (Anta-miR-17∼92) or antagomirs with negative control sequence (Anta-NC) and cultured for 48 h; or then, the normal culture medium (glucose, 5 mM) was replaced by high glucose-containing medium (glucose, 25 mM), cultured for 24 h. ^∗^*p* < 0.05, vs. the control group; ^#^*p* < 0.05, vs. the HG+Anta-NC group.

As displayed in Figure 2(a), HG stimulation significantly increased the expression of the miR-17∼92 cluster members in MPC5. Transfection with the miR-17∼92 cluster antagomirs markedly inhibited the expression of miR-17-5p, miR-18a, miR-19a, miR-19b, miR-20a, and miR-92a in MPC5, compared with the Anta-NC transfection group (Figure 2(a)). Besides, HG-treated cells signally inhibited the cell viability and induced cell apoptosis in MPC5 (Figures 2(b) and 2(c)). At the same time, the levels of IL-6 and TNF-*α* were markedly increased in HG-treated MPC5 cells (Figures 2(d) and 2(e)). Whereas silence of the miR-17∼92 cluster notably suppressed the decline of cell viability, it increased cell apoptosis and expression of proinflammatory cytokines (IL-6, TNF-*α*) in HG-treated MPC5 cells (Figures 2(b)–2(e)). These results suggested that the inhibition of the miR-17∼92 cluster enhanced the cell viability and inhibited the cell apoptosis and inflammation in HG-stimulated MPC5 cells.

### 3.3. The Effect of miR-17∼92 Cluster Downregulation on Fibrosis and Dysfunction of Podocytes

**Figure 3:**
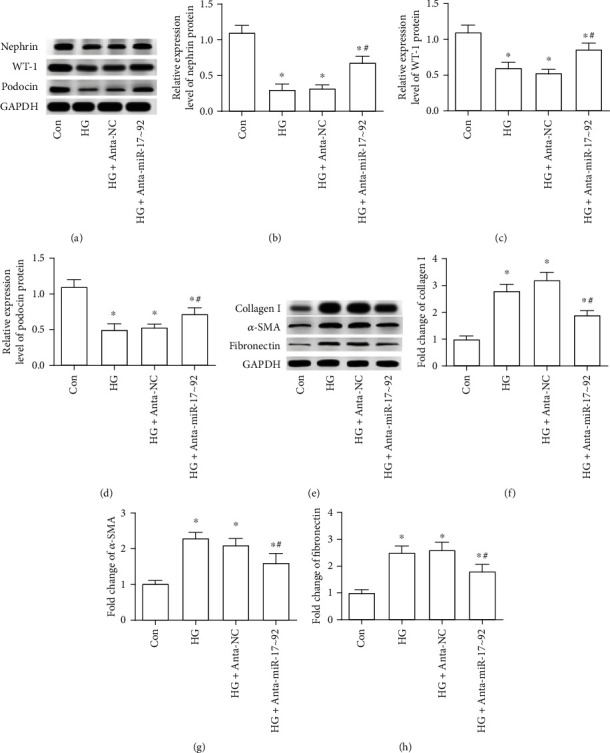
The effect of miR-17∼92 cluster downregulation on fibrosis and dysfunction of podocytes. (a–d) The expression of podocyte-specific proteins nephrin, WT-1, and podocin was measured by Western blotting. (e–h) The protein expression of collagen I, *α*-SMA, and fibronectin in MPC5 cells was tested through Western blotting. ^∗^*p* < 0.05, vs. the control group; ^#^*p* < 0.05 vs. the HG+Anta-NC group. Cell treatment was the same as described in Figure 2.

Further, the effect of miR-17∼92 cluster downregulation on fibrosis and dysfunction of podocytes were explored in this study. As displayed in Figures 3(a)–3(d), the expression of podocyte functional markers, nephrin, WT-1 (Wilms' tumor protein), and podocin was significantly decreased in HG-treated MPC5, while the downregulation of the miR-17∼92 cluster partly inhibited this decline (Figures 3(a)–3(d)). In addition, the protein expression of collagen I, *α*-smooth muscle actin (*α*-SMA), and fibronectin were significantly increased in HG-stimulated MPC5 cells (Figures 3(e)–3(h)), whereas the downregulation of the miR-17∼92 cluster effectively suppressed the increase of collagen I, *α*-SMA, and fibronectin that was induced by HG treatment (Figures 3(e)–3(h)). It is indicated that the downregulation of the miR-17∼92 cluster effectively suppressed podocyte dysfunction and fibrosis induced was by HG treatment.

### 3.4. ABCA1 Is a Potential Effector of the miR-17-92 Cluster

**Figure 4:**
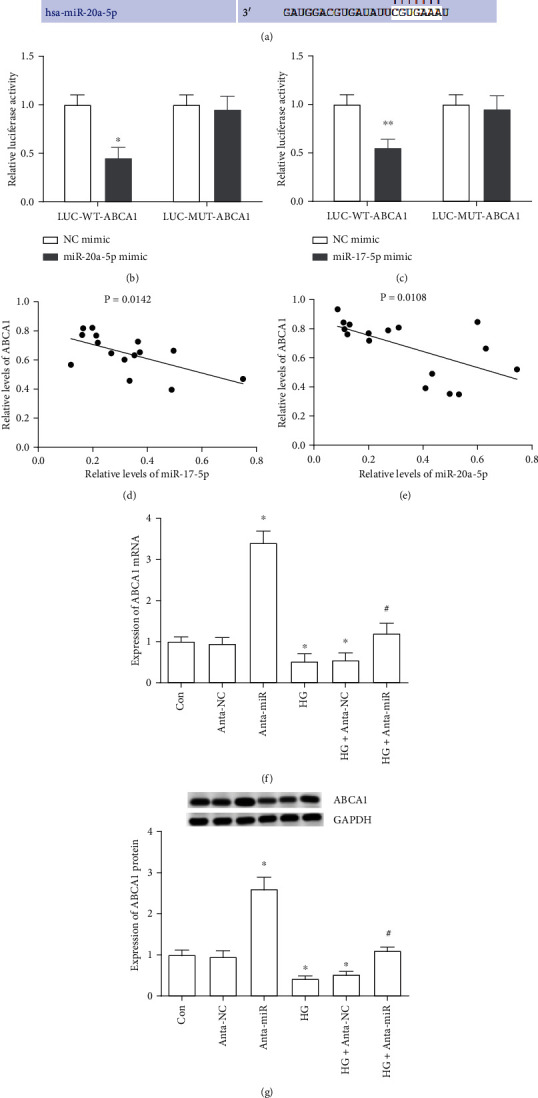
ABCA1 is a potential effector of the miR-17~92 cluster. (a) ABCA1 mRNA contains a site complementary to miR-17-5p and miR-20a-5p in the 3′UTR region. (b, c) Luciferase activities of WT-ABCA1-3′UTR and MUT-ABCA1-3′UTR luciferase reporters after being transfected with miR-17-5p mimics (b) or miR-20a-5p mimics (c) were examined through the Dual-Luciferase Reporter Assay Kit. ^∗^*p* < 0.05, vs, the NC-mimic transfection group. (d, e) The correlation among miR-17-5p, miR-20a-5p, and ABCA1 expression levels in the renal tissue samples (*n* = 15). (f, g) The expression of ABCA1 mRNA and protein was examined through RT-qPCR and Western blotting. In the Anta-miR group, cells were transfected with antagomirs of miR-17-5p and miR-20a-5p. ^∗^*p* < 0.05, vs. the control group or Anta-NC group; ^#^*p* < 0.05, vs the HG+Anta-NC group.

Bioinformatics databases TargetScan and miRbase programs were used to predict the putative miR-17~92 target genes. We identified ABCA1 as a new target of miR-17~92, containing a highly conserved binding site for miR-17-5p and miR-20a-5p in the 3′UTR region (Figure 4(a)). To address whether binding of miR-17-5p and miR-20a-5p to ABCA1 mRNA leads to its translational suppression, 3′UTR of the mouse ABCA1 gene was cloned into the luciferase reporter vector. Meanwhile, a mutant putative binding site (GCACUUUA mutated into GCCCCCUA) was generated and cloned into the luciferase reporter vector (Figure 4(a)). The results suggested that cotransfection of miRNA-17-5p or miR-20a-5p mimics with LUC-WT-ABCA1 3′UTR markedly suppressed the luciferase activity in MPC5 cells, while cotransfection of miRNA-17-5p or miR-20a-5p mimics with LUC-MUT-ABCA1 3′UTR did not have significant effects on the expression of luciferase (Figures 4(b) and 4(c)). In addition, we found that the ABCA1 expression was negatively related with miR-17-5p and miR-20a-5p levels in renal tissues of DN patients (Figures 4(d) and 4(e)). Further, HG-treated cells markedly inhibited the expression of ABCA1 (Figures 4(f) and 4(g)), while miR-17-5p and miR-20a-5p inhibition significantly promoted the expression of ABCA1 in both mRNA and protein levels in MPC5 cells and HG-treated MPC5 cells (Figures 4(f) and 4(g)). These results indicated that ABCA1 is a potential effector of the miR-17~92 cluster, and its translation was suppressed by miR-17-5p and miR-20a-5p.

### 3.5. The Effect of ABCA1 Overexpression on the Cell Viability, Apoptosis, and Function of HG-Treated MPC5 Cells

**Figure 5:**
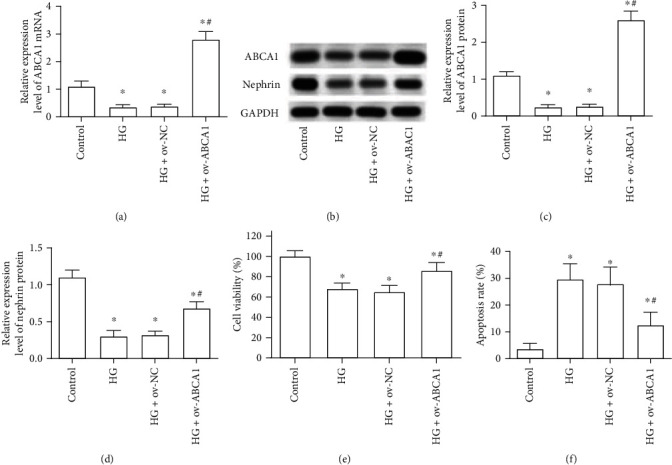
The effect of ABCA1 overexpression on the cell viability, apoptosis, and function of HG-treated MPC5 cells. (a). The expression of ABCA1 mRNA was examined after transfection with overexpression vectors of ABCA1. (b–d) The expression of ABCA1 (b, c) and nephrin (b, d) were detected through Western blotting. (e, f) Cell viability (e) and apoptosis (f) in MPC5 cells were detected through CCK-8 assay and Annexin V/PI double stain assay. ^∗^*p* < 0.05, vs. the control group; ^#^*p* < 0.05, vs. the HG+ov-NC group.

As shown in Figures 5(a) and 5(b), transfection with the overexpression vector of ABCA1 significantly suppressed the decline of ABCA1 mRNA and protein in HG-treated MPC5 cells. Then, we found that the overexpression of ABCA1 effectively inhibited the decline of cell viability, the increase of cell apoptosis, and the decline of the expression of nephrin that are induced by HG in MPC5 cells (Figures 5(c)–5(f)).

### 3.6. Silence of ABCA1 Abolished the Effect of si-miR-17∼92 Cluster on Cell Viability, Cell Apoptosis, Inflammation, Podocyte Dysfunction, and Fibrosis

**Figure 6:**
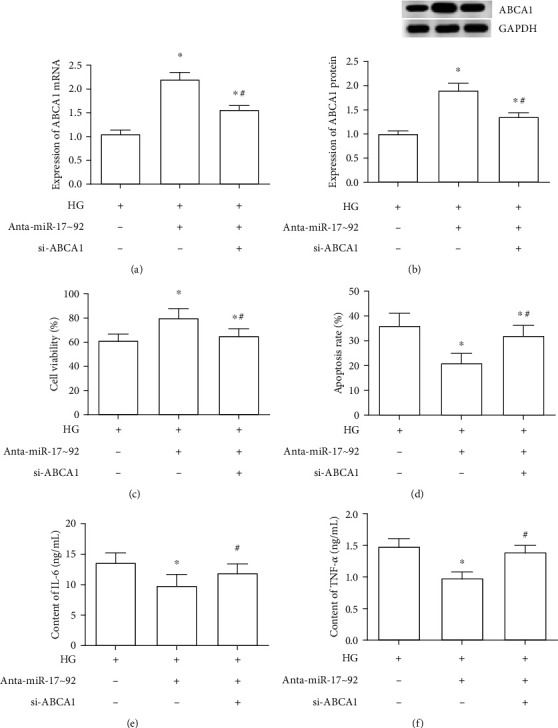
Silence of ABCA1 abolished the effect of miR-17∼92 cluster inhibition on cell viability, apoptosis, and inflammation. The expression of ABCA1 mRNA (a) and protein (b) was examined after cotransfection with miR-17~92 antagomirs and si-ABCA1. Cell viability (c), apoptosis (d), content of IL-6 (e), and TNF-*α* (f) in MPC5 cells were detected through CCK-8 assay, Annexin V/PI double stain assay, and ELISA kit, respectively. ^∗^*p* < 0.05, vs. the HG-treated group. ^#^*p* < 0.05, vs. the HG+Anta-miR-17∼92 group.

**Figure 7:**
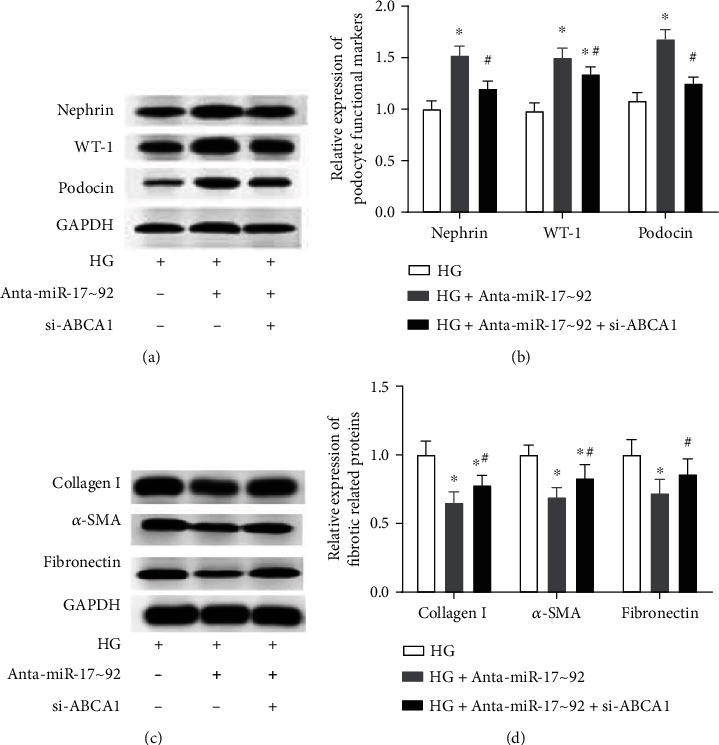
Silence of ABCA1 reversed the effect of miR-17∼92 antagomirs on fibrosis and dysfunction of podocytes. (a, b) Expression levels of nephrin, WT-1, and podocin in MPC5 cells were detected through Western blotting. (c, d) Expression levels of collagen I, *α*-SMA, and fibronectin in MPC5 cells were measured by Western blotting. ^∗^*p* < 0.05, vs. the HG-treated group. ^#^*p* < 0.05, vs. the HG+Anta-miR-17∼92 group.

To further provide evidence that ABCA1 is an effector of the miR-17∼92 cluster. si-ABCA1 and miR-17∼92 cluster antagomirs were cotransfected into MPC5 cells. We found that si-ABCA1 effectively inhibited the promoting effect of miR-17∼92 cluster antagomirs on the expression of ABCA1 in HG-treated MPC5 cells (Figures 6(a) and 6(b)). Besides, ABCA1 knockdown significantly reversed the remission effect of miR-17∼92 antagomirs on the declined cell viability induced by HG in MPC5 cells (Figure 6(c)). At the same time, ABCA1 downregulation abrogated the inhibitory effect of miR-17∼92 antagomirs on cell apoptosis, inflammation, podocyte dysfunction, and fibrosis in HG-treated MPC5 cells (Figures 6(d)–6(f) and 7(a)–7(d)).

## 4. Discussion

miRNAs play important roles in kidney development in embryonic stages; the absence of miRNAs in the early metanephric mesenchyme induced loss of nephron progenitors and caused severe renal dysgenesis [29]. Accumulating evidences suggest that some miRNAs were dysregulated in DN and participated in podocyte differentiation, homeostasis, hyperglycemia, inflammation, and fibrosis [29]. Our study proved that the miR-17∼92 cluster members miR-17-5p, miR-18a, miR-19b, and miR-20a were upregulated in the plasma and kidney tissue of DN patients. Another important finding of our study is that the expression of miR-19a and miR-92a was decreased in the plasma and increased in the kidney tissue of DN patients. This result was supported by previous study that miR-19a and miR-92a were downregulated in diabetes [17, 18], while upregulated in kidney tissues of chronic kidney disease [30, 31]. And another study indicated that the miR-17∼92 cluster is increased in a mouse model of polycystic kidney disease [19]. In addition, it has been reported that the expression of miR-92a was declined in platelets of type 2 diabetes mellitus [32] and increased in the diabetic rat pancreas [33]. Hence, our study suggested that miR-19a and miR-92a exhibited tissue specificity.

Further, our study indicated that miR-17∼92 cluster members miR-17-5p and miR-20a-5p directly target ABCA1 and that the downregulation of miR-17∼92 alleviated the podocyte injury induced by HG treatment. ABCA1, as a sterol-induced membrane protein, mainly mediates the cholesterol export [21]. Previous reports have suggested that ABCA1-mediated cholesterol efflux in podocytes is impaired, and the overexpression of ABCA1 could reduce albuminuria in mice [34, 35]. Besides, ABCA1 recycling disorders under hyperglycemia further aggravated cellular injury in podocytes [36]. Deleting ABCA1 lead to inflammation, impaired *β*-cell function, mitochondrial dysfunction in podocytes, and rendered mice susceptible to diabetic kidney disease [37–39]. In our study, we found that overexpression of ABCA1 effectively reversed the podocyte damage induced by HG treatment.

It is known that podocyte damage is accompanied with the decline of cell viability, and the increase of cell apoptosis, inflammation, fibrosis, and podocyte dysfunction. In our study, inflammatory cytokines IL-6 and TNF-*α* were increased in high glucose-cultured MPC5 cells, which is consistent with those previously reported [28], while miR-17-5p and miR-20a-5p inhibition partly suppressed this increase through upregulating ABCA1. Besides, miR-17~92 inhibition suppressed the increase of podocyte functional markers nephrin, WT-1, and podocin in HG-treated MPC5 cells. Nephrin, as a transmembrane protein, is located at the slit diaphragm of podocytes. It is indispensable in the kidney glomerular filtration barrier function [40]. It was reduced in the glomeruli of DN patients [41]. Podocin is encoded by the NPHS2 gene; its deletion leads to familial focal segmental glomerulosclerosis [41]. Meanwhile, podocin directly interacts with nephrin, regulating the glomerular permeability [42]. WT-1 is as a zinc-finger transcription factor, playing a crucial role in the nephrogenesis and the differentiation of podocytes. It has great value on the diagnosis of podocyte lesion [43]. Defects in WT-1 are the cause of multiple renal diseases, such as nephrotic syndrome type 4, Denys-Drash syndrome, and Frasier syndrome [44–46]. What is more, fibrotic-related proteins *α*-SMA, fibronectin, and collagen I were increased in HG-treated MPC5 cells, which is consistent with previous reports [47]. Experimental studies also showed that knockdown of miR-17∼92 significantly impeded the progress of fibrosis in podocytes. In summary, our study suggested that miR-17∼92 antagomirs play a protective role in HG-induced podocyte injury through regulating the expression of ABCA1.

## 5. Conclusion

The miR-17∼92 cluster was upregulated in the renal biopsy tissue of diabetic nephropathy patients; inhibition of the miR-17∼92 cluster suppressed the increase of cell apoptosis, inflammation, fibrosis, and podocyte dysfunction induced by high-glucose culture. ABCA1 is a potential effector of the miR-17∼92 cluster; binding with miR-17-5p and miR-20a, it was declined in the HG-treated MPC5 cells. Our study indicated that miR-17∼92 antagomirs ameliorates podocyte injury.

## Data Availability

Yes, if needed.
